# Computational identification of multi-omic correlates of anticancer therapeutic response

**DOI:** 10.1186/1471-2164-15-S7-S2

**Published:** 2014-10-27

**Authors:** Lindsay C Stetson, Taylor Pearl, Yanwen Chen, Jill S Barnholtz-Sloan

**Affiliations:** 1 Case Comprehensive Cancer Center; 2 Center for Proteomics and Bioinformatics, Case Western Reserve University, Cleveland, Ohio 44106 USA; 3 Department of Biological Engineering, Massachusetts Institute of Technology, Cambridge, Massachusetts 02139 USA

## Abstract

**Background:**

A challenge in precision medicine is the transformation of genomic data into knowledge that can be used to stratify patients into treatment groups based on predicted clinical response. Although clinical trials remain the only way to truly measure drug toxicities and effectiveness, as a scientific community we lack the resources to clinically assess all drugs presently under development. Therefore, an effective preclinical model system that enables prediction of anticancer drug response could significantly speed the broader adoption of personalized medicine.

**Results:**

Three large-scale pharmacogenomic studies have screened anticancer compounds in greater than 1000 distinct human cancer cell lines. We combined these datasets to generate and validate *multi-omic *predictors of drug response. We compared drug response signatures built using a penalized linear regression model and two non-linear machine learning techniques, random forest and support vector machine. The precision and robustness of each drug response signature was assessed using cross-validation across three independent datasets. Fifteen drugs were common among the datasets. We validated prediction signatures for eleven out of fifteen tested drugs (17-AAG, AZD0530, AZD6244, Erlotinib, Lapatinib, Nultin-3, Paclitaxel, PD0325901, PD0332991, PF02341066, and PLX4720).

**Conclusions:**

*Multi-omic *predictors of drug response can be generated and validated for many drugs. Specifically, the random forest algorithm generated more precise and robust prediction signatures when compared to support vector machines and the more commonly used elastic net regression. The resulting drug response signatures can be used to stratify patients into treatment groups based on their individual tumor biology, with two major benefits: speeding the process of bringing preclinical drugs to market, and the repurposing and repositioning of existing anticancer therapies.

## Background

A major challenge in precision medicine is the transformation of *multi-omic *data into knowledge that enables stratification of patients into treatment groups based on predicted clinical response. Some progress has been made to associate genetic lesions and expression profiles with drug response. The link between a patient's therapeutic response and somatic alterations in the cancer genome was established by the National Cancer Institute (NCI) using the NCI60 human tumor cell line anticancer drug screen [1]. The analysis done by the NCI led to the discovery that mutations in *BRAF *and *EGFR *are highly predictive of clinical response to kinase inhibitors [2,3]. Recently, the use of imatinib to selectively target the protein product of the *BCR-ABL *translocation revolutionized treatment of chronic myeloid leukemia [4]. Nevertheless, many cancer drugs have yet to be linked to the biomarkers necessary for assessing the effectiveness of the proposed therapeutic intervention.

Using *multi-omic *data to develop a statistical model predictive of drug response is not a trivial task. Single gene alterations discovered by linear regression techniques are often false-positive discoveries that mask the underlying biological pathway dysregulation driving drug response. There remains an urgent need to use multivariate and non-linear statistical methods to build robust *multi-omic *predictors of drug response that incorporate information from a myriad of biological alterations.

Although clinical trials remain the only way to truly measure drug toxicities and effectiveness, as a scientific community we lack the resources to clinically assess all drugs presently under development. Therefore, there is great enthusiasm to develop a preclinical system that would allow for high-throughput testing of cancer cell lines against large numbers of drug compounds in parallel. Preclinical computational models predictive of the drug response could be built based on genomic and drug screening results. Drug response signatures could be confirmed using independent validation datasets and patient tumor samples. We acknowledge that biological findings in cell lines and animal model systems have not always validated in human tumors. However, successfully validated drug response signatures have the potential to significantly speed the personalized matching of drugs to patient based on the patient's unique tumor biology.

In March 2012, the results of two large-scale pharmacogenomic human cancer cell line screens were published in *Nature *[5,6]. The Cancer Cell Line Encyclopedia (CCLE), published by researchers at the Broad Institute, and the Cancer Genome Project (CGP), presented by scientists at the Sanger Institute, complement the existing NCI60 pharmacogenomic database. Analyzing these databases in tandem potentiates the discovery of powerful, independently validated biomarkers of drug response. In this study, we used the NCI60, CCLE, and CGP pharmacogenomic datasets and evaluated the effectiveness of different computational approaches in deriving *multi-omic *signatures predictive of drug response. To our knowledge, this is the first time that all three datasets have been analyzed in a single study. A previous study attempted to develop genomic predictors of drug response using only gene expression data from the CCLE and CGP datasets [7]. Here we present an integrative analysis of high-throughput genomic and transcriptomic data; the resulting *multi-omic *signatures of therapeutic drug response have been validated across independent datasets. Using non-linear machine learning techniques, we generated robust *multi-omic *signatures that predict cellular response to 17-AAG, AZD0530, AZD6244, Erlotinib, Lapatinib, Nultin-3, Paclitaxel, PD0325901, PD0332991, PF02341066, and PLX4720.

## Materials

To develop *multi-omic *predictors of anticancer therapeutic response we curated data from the CCLE, CGP, and NCI60 databases. The resulting datasets consisted of the gene expression (Affymetrix U133A and Affymetrix U133A plus 2.0), copy number variation (Affymetrix SNP6.0), and mutational status (targeted and whole exome sequencing) of 1299 distinct human cancer cell lines representing 35 cancer types. Our curated data also included the sensitivity of the cell lines to treatment with fifteen drugs common across the CGP and CCLE databases (see Table 1). This publicly available data can be downloaded at http://broadinstitute.org/ccle (CCLE), http://cancerrxgene.org (CGP), and http://discover.nci.nih.gov/cellminer/ (NCI60).

**Table 1:** Common drugs in the Cancer Genome Project (CGP) and Cancer Cell Line Encyclopedia (CCLE) datasets

Compound	Target(s)	Class	Organization
17-AAG*	HSP90	Targeted other	Bristol-Myers Squibb
AZD0530	Src, Abl, EGFR	Kinase Inhibitor	AstraZeneca
AZD6244*	MEK	Kinase Inhibitor	AstraZeneca
Crizotinib*	c-MET, ALK	Kinase Inhibitor	Pfizer
Erlotinib*	EGFR	Kinase Inhibitor	Genentech
Lapatinib*	EGFR, HER2	Kinase Inhibitor	GlaxoSmithKline
Nilotinib*	Abl/Bcr-Abl	Kinase Inhibitor	Novartis
Nutlin-3*	MDM2	Targeted other	Roche
NVP-TAE684*	ALK	Kinase Inhibitor	Novartis
Paclitaxel*	Beta-Tubulin	Cytotoxic	Bristol-Myers Squibb
PD-0325901	MEK	Kinase Inhibitor	Pfizer
PD-0332991	CDK4/6	Kinase Inhibitor	Pfizer
PHA-665752	c-MET	Kinase Inhibitor	Pfizer
PLX4720	RAF	Kinase Inhibitor	Plexxikon
Sorafenib*	FLT3, c-KIT, PDGFR, RET, Raf kinases, VEGFR, KDR, FLT4	Kinase Inhibitor	Bayer

* Indicates compound found in CGP, CCLE and NCI60 datasets

### Drug sensitivity

Cellular drug sensitivity was measured as the concentration of drug, in micromolar units (µM), needed to inhibit 50% of cellular growth (IC_50_). The drug compounds were robotically added to cell cultures and after 72 hours cell viability was assessed by measuring the ATP content of the assay [further details can be found in the supplementary methods of 5, 6]. In our analysis, models were trained using the 10% most sensitive and resistant cell lines (lowest and highest IC_50_) to each drug of interest. The utilization of the cell lines at the limits of drug response increased the likelihood of identifying the *multi-omic *features that drive drug response.

### Gene expression

Raw gene expression CEL files were normalized using fRMA [8] and R Bioconductor [9] chip packages ('hthgu133a' for CGP and NCI60; 'hgu133plus' for CCLE). Probesets not common across the datasets were discarded. Batch effects within and between the CCLE, CGP, and NCI60 datasets were removed using the ComBat function from the R 'sva' package [10]. Finally, for each Entrez gene ID the R package 'jetset' [11] was used to select the best probeset for each gene such that each gene is represented by one probe (12,151 genes).

### Copy number variation

Copy number segments for 426 cancer genes were predicted using the PICNIC algorithm [12]. The raw CNV values were converted into five categories: amplification (copy number of eight or more), partial amplification (copy number between three and seven), normal (copy number of two), partial deletion (copy number of one), and full deletion (copy number of zero).

### Mutational status

The mutational status of sixty-four commonly mutated cancer genes was assessed. A gene was defined as mutated if a coding sequence variant was present. Additionally, for cell lines in the CGP and CCLE databases the presence or absence of commonly rearranged cancer genes was determined.

## Methods

**Figure 1:**
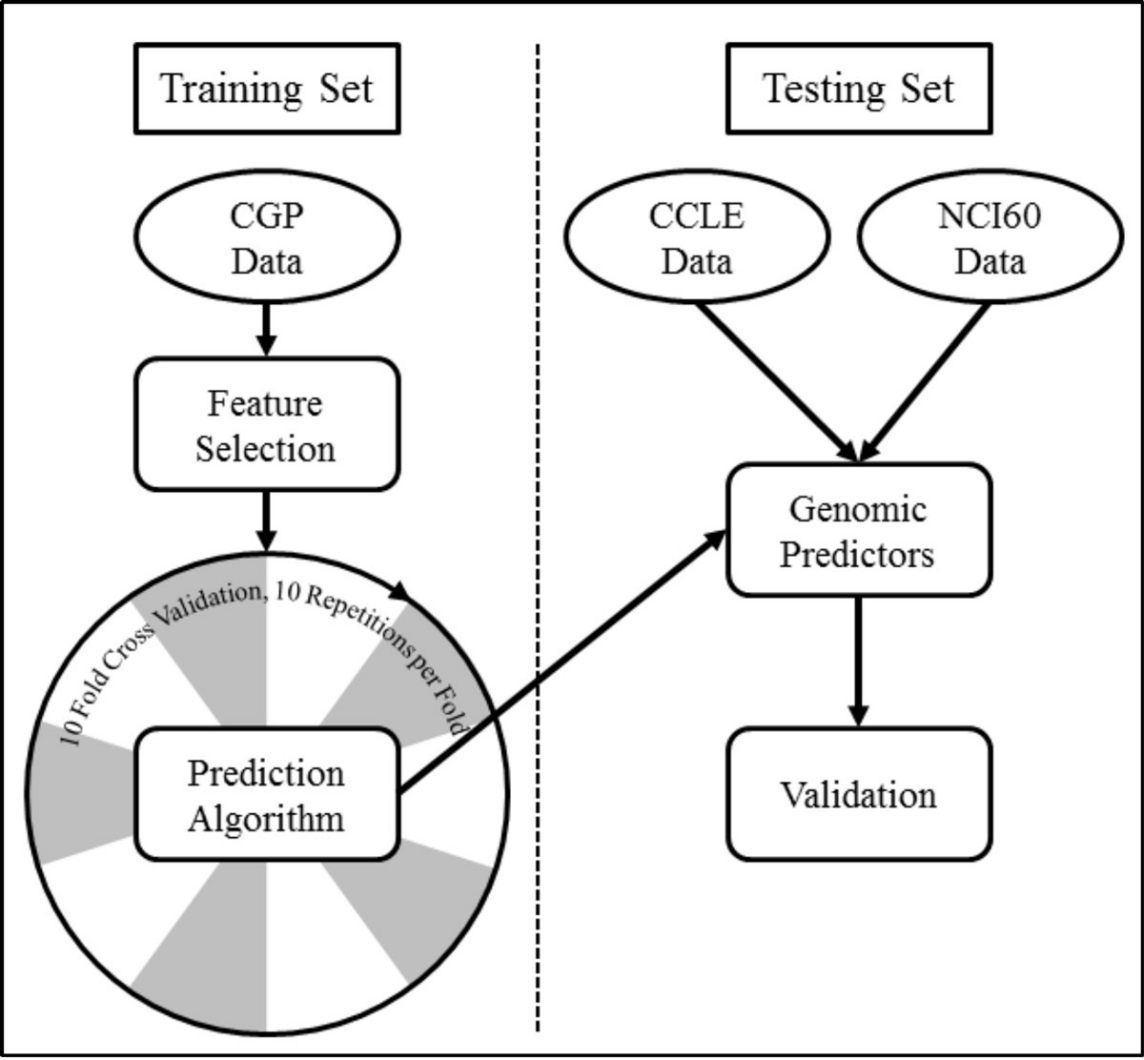
**Experimental approach included subjecting the Cancer Genome Project (CGP) training data to statistical feature selection and training each machine learning algorithm with the resulting feature subset**. The resulting genomic predictors of drug response to 15 anticancer drugs of interest were validated on independent Cancer Cell Line Encyclopedia (CCLE) and National Cancer Institute (NCI60) datasets.

The approach used to build *multi-omic *signatures predictive of drug response is illustrated in Figure 1. The drug response signatures were generated using a two-step procedure consisting of statistical feature selection, to reduce the complexity of the datasets, followed by a classification algorithm, to weight each feature's contribution to the overall prediction. The predictive models were generated using CGP data as input and subjected to ten-fold cross-validation with ten repetitions per fold. The outputs of the process were weighted *multi-omic *drug response signatures. A signature size of thirty has previously been reported as an optimal balance between clinical relevance and genomic complexity [13,14], therefore we limited our final predictive signatures to the top thirty features. The final signatures were then tested for accuracy and over-fitting using the independent CCLE and NCI60 datasets.

### Feature selection

The feature selection inputs were as follows:

1. A matrix of features *X ∈ P ^N, p ^*, where *N *was the total number of cell lines in the CGP dataset and *p *was the number of *multi-omic *features (gene expression, CNV, and mutation).

2. A vector of drug sensitivities, *Y ∈ P ^N, 1 ^*, where N was the number of cell lines treated with the drug of interest and the vector values were the corresponding cellular drug sensitivities (refer to section "Materials: Drug sensitivity").

For each drug the Wilcoxon Sum Rank Test (for continuous variables) was used to select genes whose expression was significantly differentially expressed between the 10% most sensitive and resistant cell lines (lowest and highest IC_50_). The Fisher's Exact Test (for categorical variables) was used for each drug to select genes whose mutational status and/or CNVs significantly differed between sensitive and resistant cell lines. The resulting machine learning input sets for each drug were comprised of the 1000 most differentially expressed features (as determined by Wilcoxon and Fischer's tests). The feature selection was completed using a custom script implemented in SAS.

### Machine learning prediction

We assessed the performance of two machine learning methods, random forest and support vector machine (SVM), in generating accurate and precise *multi-omic *signatures predictive of drug response using the input sets for each drug comprised of the 1000 most differentially expressed features. We compared the performance of these models to the accuracy and precision of the drug response signature generated using elastic net regression, a type of penalized linear regression.

The random forest model was implemented in R using the 'RandomForestSRC' package [15,16]. We evaluated several combinations of forest size (*ntree*), number of features selected at each node (*mtry*), and node size (*nodesize*). The parameters resulting in the highest prediction accuracy were *ntree *= 2000*, mtry *= feature set size/3, and *nodesize *= 1. For each tree, the prediction error on the out-of-bag data was recorded. For each feature *x *the out-of-bag cases were randomly permuted and the prediction error was recorded. The variable of importance for each feature was defined as the difference between the perturbed and unperturbed error rate averaged over all trees.

A support vector machine was implemented using the 'libsvm' R package [17]. The SVM was used was a type *C classification *machine using a radial basis kernel. Features were ranked based on their weight magnitude (ω^2^)--a measure of class predictive ability.

The 'glmnet' R package was used to implement elastic net regression [18]. We selected the same optimal regularization parameters used in the primary CGP and CCLE publications: α ε (0.2, 1.0) and λ = e^γ^, where γ ε (-6, 5)--which minimized the root mean squared error using ten-fold cross-validation [5-7].

## Results

We have evaluated the effectiveness of three computational approaches for deriving clinically relevant *multi-omic *signatures predictive of drug response. In our study, we compared elastic net regression, a commonly used linear method; support vector machine; and random forest, an effective ensemble method.

**Figure 2:**
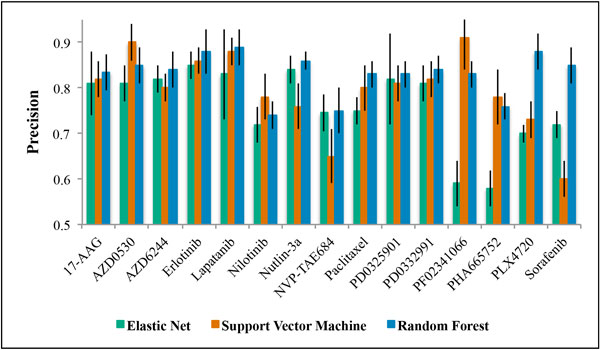
**Mean prediction performance of *multi-omic *drug response signatures generated using elastic net regression, a support vector machine, or random forest on the Cancer Genome Project (CGP) data**. Prediction performances (precision) are quantified as the proportion of true positive drug sensitive classifications to all positive classifications. Error bars represent the standard deviation of the precisions calculated during ten repetitions of ten-fold cross validation.

Signatures consisting of thirty *multi-omic *features were generated using the CGP pharmacogenomic database as a training set. The performance of these signatures in predicting drug response was assessed as precision. Precision was calculated as the ratio of true classifications of cellular drug sensitivity to all positive classification results. As illustrated in Figure 2, we observed significant and clinically relevant signature performance, precision greater than 0.80, for twelve out of fifteen drugs. The random forest approach yielded the most precise results for ten out of the twelve prediction signatures generated (17-AAG, AZD6244, Erlotinib, Lapatinib, Nultin-3, Paclitaxel, PD0325901, PD0332991, PLX4720, Sorafenib). The support vector machine approach yielded the most precise results for two out of the twelve prediction signatures generated (AZD0530 and PF02341066). Elastic net regression failed to yield a top performing prediction signature for any of the fifteen drugs evaluated.

**Figure 3:**
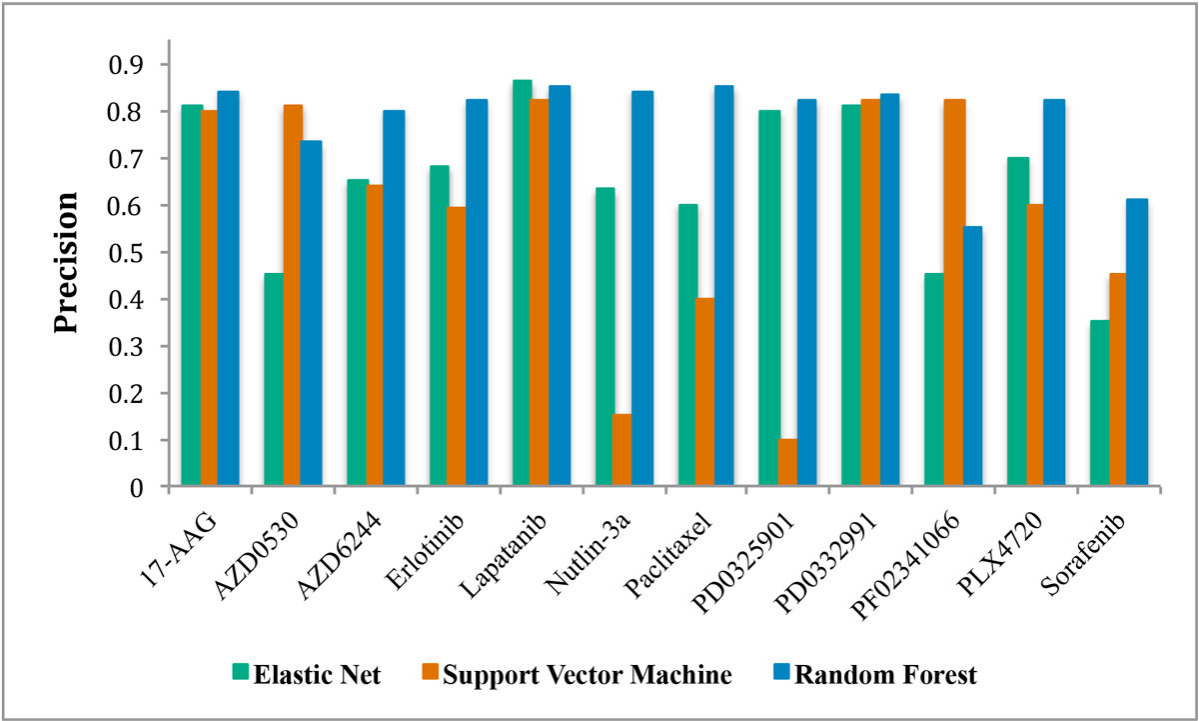
**Mean prediction performance of *multi-omic *drug response signatures generated using elastic net regression, a support vector machine, or random forest on the Cancer Genome Project (CGP) data and tested/validated on the Cancer Cell Line Encyclopedia (CCLE) data**.

Independent validation of our generated signatures was performed using the CCLE and NCI60 datasets. As illustrated in Figure 3, eleven of the twelve drug response signatures developed using the CGP dataset successfully predicted, with a precision greater than 0.80, drug response in the CCLE dataset (17-AAG, AZD0530, AZD6244, Erlotinib, Lapatinib, Nultin-3, Paclitaxel, PD0325901, PD0332991, PF02341066, and PLX4720). Using the NCI60 dataset we were able to predict drug response, with a precision greater than 0.80, for seven out of eight signatures for drugs that are common across the CGP and NCI60 databases (17-AAG, AZD6244, Erlotinib, Lapatinib, Nultin-3, Paclitaxel, and PF02341066).

## Discussion

Significant computational effort has been expended in the past ten years to build robust and clinically relevant predictors of drug response. Preclinical efforts have previously been limited by the lack of publically available data from which to build prediction sets. The publication of the CGP and CCLE pharmacogenomics datasets in March of 2012 made large-scale integrated analysis of *multi-omic *and drug response data possible [5,6]. To our knowledge, the genomic, transcriptomic, and drug profiling data contained in the CGP, CCLE, and NCI60 databases has not previously been analyzed in concert. We have combined these three datasets to generate and independently validate genomic correlates of anticancer drug response.

The purpose of our study was twofold: to show that precise and robust predictors of drug response could be built and to explore the use of multivariate linear and non-linear statistical methods in building the predictors. Previous studies have relied heavily upon commonly used penalized linear regression models in developing predictive genomic signatures, likely because of the challenges inherent to machine learning techniques[5-7,19]. While machine learning algorithms such as random forest and support vector machine can be more computationally intense and difficult to interpret, our work shows that the signatures derived there from have superior prediction power and robustness [15,20-23]. Non-linear machine learning algorithms are more effective in generating prediction signatures, as opposed to linear regression, because they make no distributional assumptions about the predictor variables and allow all features, including those with individually weak effects, to contribute to the model fit.

We generated *multi-omic *predictors of drug response to fifteen drugs of interest. During the signature-generation phase we created and validated predictive signatures using the CGP dataset (Figure 2). Using elastic net regression, eight of the fifteen signatures successfully predicted drug response with a precision greater than 0.80 (17-AAG, AZD0530, AZD6244, Erlotinib, Lapatinib, Nultin-3, PD0325901, PD0332991). Using a support vector machine, nine of the fifteen signatures successfully predicted drug response with a precision greater than 0.80 (17-AAG, AZD0530, AZD6244, Erlotinib, Lapatinib, Paclitaxel, PD0325901, PD0332991, PF02341066). The random forest algorithm was the most powerful approach. Using random forest, twelve of the fifteen signatures successfully predicted drug response with a precision greater than 0.80 (17-AAG, AZD0530, AZD6244, Erlotinib, Lapatinib, Nultin-3, Paclitaxel, PD0325901, PD0332991, PF02341066, PLX4720, Sorafenib).

We were unable to generate predictive signatures for three of the fifteen drugs of interest: Nilotinib, NVP-TAE684, and PHA665752 (Figure 2). NVP-TAE684 and Nilotinib target the protein products of gene fusions, *NPM-ALK *and *BCR-ABL *respectively. These gene fusions were not well represented in our datasets, making signature generation difficult. The low number of cell lines in the datasets sensitive to PHA665752 contributed to the difficulty of generating a predictive signature with good precision for this drug. While a signature could not be generated for PHA665752 reaching our precision cutoff of 0.80, the random forest and support vector machine signatures, with precisions of 0.76 and 0.78, greatly outperformed elastic net regression, which achieved a precision of 0.58. The performance of the non-linear algorithms was markedly superior to that of the linear regression algorithm when N, the number of cell lines sensitive to the drug of interest, was very small in comparison to p, the total number of *multi-omic *features.

The predictive performance of the *multi-omic *signatures was tested against the CCLE and NCI60 datasets for robustness (Figure 3). Only 50% of the signatures generated using elastic net regression and support vector machine could be validated on independent datasets. In comparison, 75% of the signatures generated using random forest were validated on independent datasets. Four out of the eight signatures developed using elastic net regression retained predictive precision greater than 0.80 when tested on the CCLE dataset (17-AAG, Lapatinib, PD0325901, PD0332991). Five out of the nine signatures developed using support vector machine retained predictive precision greater than 0.80 when tested on the CCLE dataset (17-AAG, AZD0530, Lapatinib, PD0332991, PF02341066). Random forest yielded more, and more robust predictive signatures, with nine out of the twelve signatures generated remaining predictive when tested against the CCLE dataset (17-AAG, AZD6244, Erlotinib, Lapatinib, Nultin-3, Paclitaxel, PD0325901, PD0332991, PLX4720). Response to the drug Sorafenib could not be independently validated using any of the generated signatures. Sorafenib is a multi-kinase inhibitor and it is likely that limiting our signatures to thirty features each did not allow enough genomic complexity to predict response to this drug.

### Random forest signatures

The random forest algorithm identified high expression of *NQO1 *as the single most important and robust predictor of a cell's sensitivity to 17-AAG, an HSP90 inhibitor. Oncogenic proteins such as Raf-1 and p53 are kept in an apoptosis resistant state by direct association with HSP90; heat shock protein inhibitors such as 17-AAG break the direct association between HSP90 and apoptotic proteins. *NQO1 *is a member of the NAD(P)H dehydrogenase quinone family and produces the highly potent and stable intermediate 17-AAGH2 when the compound 17-AAG is metabolized [24]. High expression of *NQO1 *has been previously reported in biological studies as a biomarker of sensitivity to 17-AAG [5-7,25].

The predictive signature of response to PD-0325901 generated by random forest confirmed several previously identified biomarkers and identified several novel biomarkers. PD-0325901, a *MEK *inhibitor, directly affects *MAPK *signaling. Given the large number of genes involved in the *MAPK *pathway, it is not surprising that the non-linear random forest approach best captured the many interacting genes that predict PD-0325901 response. The random forest approach confirmed previous knowledge indicating that high expression of *SPRY2, LGALS3*, and *PHLDA1 *predict sensitivity to PD-0325901: *SPRY2 *suppresses cell growth and differentiation by inhibiting the *MAPK *pathway [26]; *LGALS3 *modulates cell-cell and cell-matrix interactions [27]; and *PHLDA1 *is known to play an important role regulating anti-apoptotic effects [28]. The random forest approach confirmed this knowledge by highly weighting these genes in the predictive signature. Our study offers the novel finding that expression of *GSN, PHF17*, and *ZFP30 *is involved in conferring cellular sensitivity to PD-0325901: *GSN *is involved in the assembly and disassembly of actin filaments needed for cellular replication [29]; *PHF17 *is a known tumor suppressor and promotes apoptosis [30]; and *ZFP30 *is thought to be involved in regulating transcription.

The random forest algorithm was uniquely successful in generating predictive signatures for cytotoxic drugs, a result that was not achieved using linear regression[7]. In the case of the broadly acting taxane drug Paclitaxel, the random forest generated signature was precise and robust. Paclitaxel sensitivity is predicted by up regulation of and amplification in the genes *ANXA1, SSRP1, PAFAH1B2*, and *PSMG3*: *ANXA1 *inhibits the activation of NF-kB by binding to p65 [31]; *SSRP1 *forms the chromatin transcription elongation factor FACT which is crucial to the anticancer mechanism of DNA damaging drugs [32]; and *PSMG3 *is a proteasome assembly chaperone [33].

### A case study: triple negative breast cancer

Triple negative breast cancer (TNBC) is defined by the absence of detectable estrogen and progesterone receptors and the lack of amplification in the human epidermal growth factor receptor 2 gene [34]. Triple negative breast cancer accounts for 15-20% of all breast cancers, is generally more aggressive, and patients have decreased overall survival [35]. TNBC does not represent a single type of breast cancer, but rather a heterogeneous group of tumors with distinct molecular subtypes. The triple negative subgroups respond differentially to drug therapy. The current standard of care for TNBC is treatment with taxanes and other cytotoxic compounds. While overall response to taxane treatment is 28%, some TNBC groups such as the luminal androgen receptor subtype have a response to taxane drugs as low as 0-10% [34]. The primary clinical problem for TNBCs is the lack of targeted therapies and a standard by which to stratify patients into the available treatments. There are currently no triple negative breast cancer drugs in phase III clinical trials, highlighting the need to identify research, repositioned, and repurposed drug compounds to treat TNBC patients [36].

We applied our random forest drug response prediction signature, generated using Cancer Genome Project (CGP) data, to TNBC cell lines in the Cancer Cell Line Encyclopedia (CCLE). We predicted that 32% of the TNBC cell lines would be sensitive to treatment with Paclitaxel. Seven out of twenty-five (28%) TNBC cell lines were true positives for sensitivity to treatment with the taxane drug Paclitaxel. This result is consistent with clinical results that indicate 28% of TNBC tumors respond to treatment with taxane drugs [34]. Additionally, we correctly predicted that a subset of triple negative breast cancer cell lines would be sensitive to treatment with 17-AAG. The group of TNBC cell lines with predicted and true sensitivity to 17-AAG belongs to the luminal androgen receptor subtype, a group that resists traditional treatment. As predicted there was a positive correlation between *NQO1 *expression and TNBC cellular sensitivity to 17-AAG (Pearson correlation coefficient: 0.61). The random forest generated prediction signature also correctly predicted that 50% of triple negative breast cancer cell lines would be sensitive to the *MEK *inhibitor PD-0325901. The sensitive cell lines roughly correspond to the basal triple negative breast cancer subtype.

TNBC remains a challenging disease; here we have identified two promising research compounds for the treatment of TNBC. Preclinical identification of promising drug compounds, such as used in the approach described in this study, offer great promise to improve treatment of TNBC.

## Conclusions

Using the random forest algorithm and support vector machine, we were able to generate and validate robust *multi-omic *signatures that predict drug response to 17-AAG, AZD0530, AZD6244, Erlotinib, Lapatinib, Nultin-3, Paclitaxel, PD0325901, PD0332991, PF02341066, and PLX4720. The non-linear machine learning techniques random forest and support vector machine outperformed the more commonly used elastic net regression in developing precise and robust genomic predictors. Our results suggest that large pharmacogenomic databases can be used to identify the genomic correlates of anticancer drug response. The resulting classification of *multi-omic *predictors of drug response could be used to stratify patients into treatment groups based on their individual tumor biology. Prediction signatures show special promise for diseases, such as triple negative breast cancer, where there remains an urgent need to identify research or repositioned compounds that can be developed as targeted treatment for this difficult to treat patient population.

Our research could be extended in multiple ways: lineage specific predictors of drug response could be explored; further tuning of machine learning parameters could yield improved prediction results; and signature including features of the epigenome and proteome could improve drug response prediction.

## Competing interests

The authors declare that they have no competing interests.

## Authors' contributions

LCS designed the experiment, performed the bioinformatics analysis, and drafted the manuscript. TP assisted with bioinformatics analysis. YC assisted in data generation. JBS conceived of the study and revised the manuscript.
