# Erratum: Computational identification of multi-omic correlates of anticancer therapeutic response

**DOI:** 10.1186/s12864-015-1630-1

**Published:** 2015-06-30

**Authors:** Lindsay C Stetson, Taylor Pearl, Yanwen Chen, Jill S Barnholtz-Sloan

**Affiliations:** Case Comprehensive Cancer Center, Cleveland, OH USA; Center for Proteomics and Bioinformatics, Case Western Reserve University, Cleveland, OH 44106 USA; Department of Biological Engineering, Massachusetts Institute of Technology, Cambridge, MA 02139 USA

## Erratum to: BMC Genomics doi:10.1186/1471-2164-15-S7-S2

Following the publication of our recent article in *BMC Genomics* [1] we wish to acknowledge the contribution to the concept for our study provided by the previous work of Papillon-Cavanagh et al. [2] and note that we should have included a reference to their paper in the legend to Figure 1. Furthermore when discussing our findings that NQO1 is a marker for sensitivity of TNBC to 17-AAG we should have cited Papillon-Cavanagh et al. [2] as a previous paper where similar results were reported. We apologize to all affected parties.
